# Disentangling associations between vegetation greenness and dengue in a Latin American city: Findings and challenges

**DOI:** 10.1016/j.landurbplan.2021.104255

**Published:** 2021-09-23

**Authors:** Maria da Consolação Magalhães Cunha, Yang Juc, Maria Helena Franco Moraisd, Iryna Dronovae, Sérvio Pontes Ribeirof, Fábio Raphael Pascoti Bruhng, Larissa Lopes Lima, Denise Marques Sales, Olivia Lang Schultes, Daniel A. Rodriguezi, Waleska Teixeira Caiaffa

**Affiliations:** a Observatory for Urban health in Belo Horizonte, School of Medicine, Federal University of Minas Gerais, Brazil; b Pontifical Catholic University of Minas Gerais, Brazil; c Institute of Urban and Regional Development, University of California, 316 Wurster Hall, University of California, Berkeley, Berkeley, CA 94720, USA; d Belo Horizonte Municipal Health Department, Brazil; e Department of Environmental Science, Policy, and Management, Department of Landscape Architecture and Environmental Planning, University of California, Berkeley, USA; f Laboratory of Ecology of Diseases and Forests, Nucleous of Biology/NUPEB and Institute of Exact and Biological Sciences, Federal University of Ouro Preto, Brazil; g Faculty of Veterinary Medicine, Federal University of Pelotas, Brazil; h Federal Center for Technological Education of Minas Gerais, Brazil; i Department of City and Regional Planning and Institute of Transportation Studies, University of California, Berkeley, USA

**Keywords:** Dengue, Greenness, Socioeconomic vulnerability, Reemerging infectious disease, Planning and policy implications

## Abstract

Being a Re-Emerging Infectious Disease, dengue causes 390 million cases globally and is prevalent in many urban areas in South America. Understanding the fine-scale relationships between dengue incidence and environmental and socioeconomic factors can guide improved disease prevention strategies. This ecological study examines the association between dengue incidence and satellite-based vegetation greenness in 3826 census tracts nested in 474 neighborhoods in Belo Horizonte, Brazil, during the 2010 dengue epidemic. To reduce potential bias in the estimated dengue-greenness association, we adjusted for socioeconomic vulnerability, population density, building height and density, land cover composition, elevation, weather patterns, and neighborhood random effects. We found that vegetation greenness was negatively associated with dengue incidence in a univariate model, and this association attenuated after controlling for additional covariates. The dengue-greenness association was modified by socioeconomic vulnerability: while a positive association was observed in the least vulnerable census tracts, the association was negative in the most vulnerable areas. Using greenness as a proxy for vegetation quality, our results show the potential of vegetation management in reducing dengue incidence, particularly in socioeconomically vulnerable areas. We also discuss the role of water infrastructure, sanitation services, and tree cover in lowering dengue risk.

## 1 Introduction

Approximately 390 million dengue cases occur globally every year (Bhatt et al., 2013). In South America, there is a high prevalence of dengue in many urban areas. However, dengue once appeared to be under control, and recurring outbreaks make it a reemerging infectious disease. The elimination of *Aedes aegypti,* the main dengue vector, by mosquito eradication programs effectively controlled the dengue epidemic in the Americas in the 1950s and 1960s (Gubler, 2011). However, dengue returned to the Antilles in 1960 and reached South America at the end of that decade. Since then, dengue has become more intense and has been affecting mainly large urban centers in different regions (Gubler, 2011; San Martín et al., 2010; Wilcox & Colwell, 2005). Unplanned urbanization, along with ineffective mosquito control, changes in lifestyles, globalization, and international travel, have contributed to dengue’s reemergence in both endemic and non-endemic areas (Buonsenso et al., 2014; Gubler, 2011). *Aedes aegypti* is a domesticated mosquito that develops in man-made containers in the urban environment, and the mosquito’s abundance raises with urbanization (Higa, 2011; Powell & Tabachnick, 2013). Urban planning and management have the potential to reduce the risk of vector-borne diseases, through improving the built environment, green spaces, housing conditions, and sanitation (Degroote, Zinszer, & Ridde, 2018; Lee, 1994; Lindsay, Wilson, Golding, Scott, & Takken, 2017; Ogden, 2016). Studying the associations between dengue incidence and the aforementioned factors at the urban neighborhood scale is critical to identify markers of elevated risk and opportunities for intervention (Spencer, Finucane, Fox, Saksena, & Sultana, 2020), refine disease prevention guidance, and increase resident’s participation in managing natural and human habitats.

Controlling emerging and reemerging infectious diseases, such as vector-borne diseases, including dengue, requires interdisciplinary knowledge and an expanded concept of causality in epidemiology (Ellis & Wilcox, 2009). Under these principles, researchers have developed frameworks including biocomplexity (Colwell, 1998; Wilcox & Colwell, 2005) and the Eco-Bio-Social strategy (de Macêdo et al., 2021). Central to these frameworks is that environmental and socioeconomic conditions interact to create elevated risks of diseases such as dengue and the recent COVID-19. Dengue incidence rises with temperature, precipitation, and relative humidity (Xu et al., 2017). It is linked to vector biology: rising temperatures expand the spatial distribution of *Aedes aegypti* both globally (Bustamante et al., 2019) and locally (Bona, Twerdochlib, & Navarro-Silva, 2011; Degallier et al., 2003). *Aedes aegypti* is a highly domesticated mosquito that lays eggs in artificial containers such as old tires, water storage, and trash (Gubler, 1998). Therefore, socioeconomic conditions including lower household accessibility to piped water (Pedrosa et al., 2020; de Teixeira, 2008), increased domestic water storage (Cordeiro et al., 2011), irregular or no trash collection (Cordeiro et al., 2011; Honorato et al., 2014; Vargas et al., 2015), and lack of basic sanitation (Cordeiro et al., 2011; de Almeida & de Medronho, 2009), have been linked to a higher incidence of dengue. In addition, lack of effective vector control (Gubler, 2011), viral serotype, local population immunity (Marti et al., 2020; Salje et al., 2012), and human mobility (Cordeiro et al., 2011; Zhu, Liu, Tan, & Shi, 2016) have been associated with dengue incidence.

Urban areas are complex mosaics of heterogeneous environmental and socioeconomic conditions, making these areas hotspots of dengue. Fast and unplanned urbanization may be accompanied with sub-standard sanitation services and underdeveloped water infrastructures, leading to favorable conditions for *Aedes aegypti* infestation. The high population density in cities also increases exposure to mosquitos and the likelihood of dengue transmission. Together, these conditions make dengue primarily an urban disease that reemerges in large tropical urban centers at a faster frequency (Gubler, 2011). With the increasing transmission of vector-borne diseases in cities, public policy has turned to multisectoral approaches, including environmental management, expansion of sanitation services, housing improvement, and educational campaigns (Donalisio et al., 2017) to reduce disease risks. In this context, modifying the built and natural environment through vegetation management and land use planning have emerged in the urban planning sector as promising approaches to reduce the spread of vectorborne diseases such as dengue (Degroote et al., 2018; Lee, 1994; Lindsay et al., 2017; Ogden, 2016).

Vegetation management can be an effective strategy in highly urbanized areas characterized by high population density and dominance of built-up areas, where other interventions including land use control and housing improvement may be more challenging and costly. Observational studies have examined dengue incidence and vegetation within and between cities. The studies found a mixture of positive, negative, and non-linear associations (Araujo et al., 2015; Cao et al., 2017; Huang et al., 2018; Martínez-Bello, López-Quílez, & Torres Prieto, 2017; Penso-Campos, Fraga, Caldas, Sommer, & Périco, 2018; Qi et al., 2015; Sari, Adelwin, & Rinawan, 2020). These inconsistent findings can be attributed to different spatial scales of analysis, measures of vegetation, and analytical approaches. Experimental studies have found that vegetation management, including planting certain species (Jiannino & Walton, 2004) and optimizing planting configuration (Thullen, Sartoris, & Walton, 2002), can reduce mosquito populations. However, these studies (Jiannino & Walton, 2004; Thullen et al., 2002) were conducted in wetlands and did not use dengue incidence as an outcome. Therefore, the effectiveness of the proposed management strategies in reducing dengue risk in cities remains uncertain. Different vegetation types, including farmland, forests, and grassland, co-exist in cities. These vegetation types could increase or decrease the *Aedes aegypti* population due to changes in competing species (Medeiros-Sousa, Fernandes, Ceretti-Junior, Wilke, & Marrelli, 2017) and shading (Barrera, Amador, & Clark, 2006), consequently modifying dengue transmission patterns. Therefore, while local governments can employ vegetation management to reduce the risk of dengue transmission, effective ways to achieve this goal remain uncertain.

**Fig. 1:**
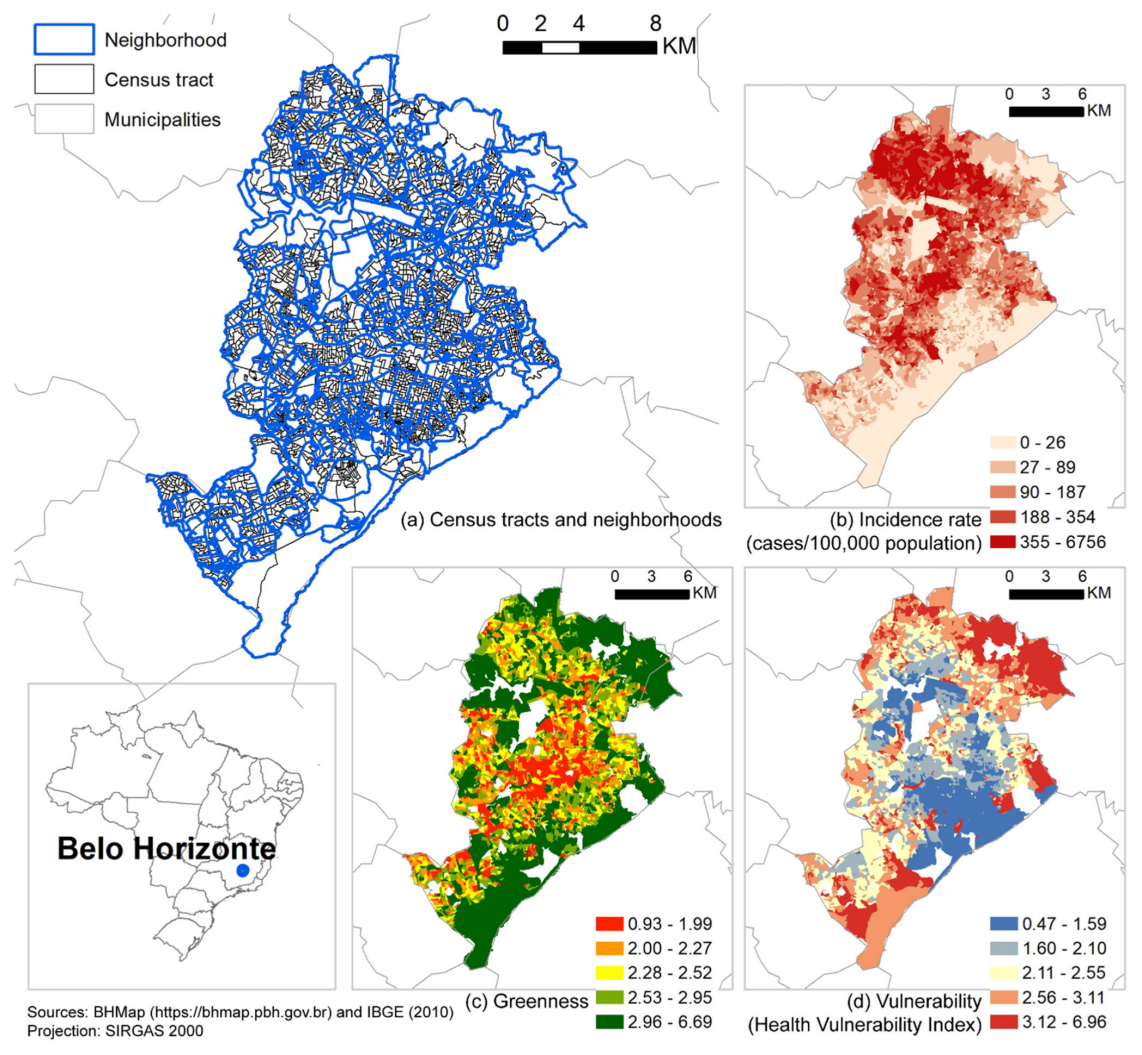
Study area. (a) Census tracts nested in neighborhoods, and spatial distribution of (b) Dengue incidence rate (cases/100,000 population) during the 2010 epidemic; (c) greenness, with higher values indicating more and better quality vegetation; (d) vulnerability, measured by the Health Vulnerability Index, with higher values indicating more vulnerability. Values in (b) - (d) are classified by quantile classification.

In this study, we examine Belo Horizonte, Brazil, which has experienced several dengue outbreaks since 1996. The city represents a complex spatial distribution of dengue cases and environmental and socioeconomic conditions (Fig. 1). Research has linked seasonal peaks in temperature and precipitation (Almeida, Assunção, Proietti, & Caiaffa, 2008; Horizonte, 2016; Campos et al., 2019), socioeconomic, demographic, and urban infrastructure characteristics (Bavia et al., 2020; Campos et al., 2019; de Mattos Almeida, Caiaffa, Assunção, & Proietti, 2007; Pessanha, Caiaffa, Kroon, & Proietti, 2010) with dengue incidence in Belo Horizonte. In 2010, twelve years after the previous epidemic, the city recorded an epidemic with the concurrent circulation of three viral serotypes (DENV-1, DENV-2, and DENV-3) and an annual incidence of 2053 cases per 100,000 inhabitants. Dengue generally exhibits a seasonal and cyclical behavior over the years due to climatic conditions, circulating viruses, and population susceptibility (Ximenes et al., 2016). Consequently, Belo Horizonte has experienced subsequent epidemics in 2013, 2016, and 2019, with progressively increasing incidence, disease severity, and fatality rates (Horizonte, 2020; Campos et al., 2019). These recurring outbreaks highlight the urgency of interventions, including those in vegetation management, to curb dengue risk.

Accordingly, to understand how vegetation management could reduce dengue risk, we investigated the association between vegetation greenness and dengue incidence in 3826 census tracts in Belo Horizonte, Brazil, during the 2010 dengue epidemic. To our knowledge, only one prior study has analyzed the relationship between dengue and vegetation at this scale (Martínez-Bello et al., 2017). This fine spatial scale is critical for communicating research findings with local decision-makers. We measured vegetation greenness using the Normalized Difference Vegetation Index (NDVI), a popular satellite-based indicator for vegetation coverage, health, and photosynthetic activities. NDVI has been widely used in environmental health studies (Rojas-Rueda, Nieu-wenhuijsen, Gascon, Perez-Leon, & Mudu, 2019).

We hypothesize that vegetation greenness has a negative association with dengue incidence, as higher greenness after controlling for vegetation coverage indicates healthier vegetation with the presence of predators and competing species of the *Aedes aegypti,* leading to a protective effect against dengue. We also explore whether the relationship between dengue incidence and greenness is modified by socioeconomic vulnerability. We hypothesize that more vulnerable areas would be at greater risk due to lower quality green spaces that may be a better habitat for the vector. To reduce potential omitted variable bias in the estimated relationships between dengue incidence and greenness, we controlled for a set of covariates, including socioeconomic vulnerability, population density, building height, percentage of census tract area covered by building footprints, land cover composition, elevation, and weather patterns. The findings are intended to inform urban planners, engineers, and public health professionals regarding vegetation management as a tool to mitigate the transmission of dengue and other arboviruses.

## 2 Data and methods

### 2.1 Study area

Our study area initially covers 3828 census tracts (setores censitários) nested in 474 neighborhoods (bairros) in Belo Horizonte, Brazil (Fig. 1). We dropped two census tracts due to missing land cover data, and we used the remaining 3826 census tracts throughout the study. As defined by the Brazilian Institute of Geography and Statistics (IBGE), a census tract is a continuous area located in a single urban or rural setting, with varying sizes and numbers of households (Bueno, 2017; IBGE, 2010). In Belo Horizonte, a census tract contains an average population of 5886 (Interquartile Range, IQR: 3800 – 7886) and an average area of 7.94 ha (IQR: 2.61 – 7.87). Neighborhood, officially defined by the city, is a cluster of adjacent census tracts by which the city communicates with its residents and provides services (Bairros de BH, 2009). We obtained neighborhood boundary data from BHGeo (https://bhgeo.pbh.gov.br/home). A neighborhood contains between 1 and 58 census tracts (Table 1).

### 2.2 Outcome: dengue incidence

The outcome variable is dengue incidence (cases per 100,000 population) in 2010. Dengue cases are reported to the Notifiable Diseases Information System, and we obtained the data for Belo Horizonte through the City Health Department. As part of the BH-VIVA database project (Friche, Dias, Reis, Dias, & Caiaffa et al., 2015), the Urban Health Observatory of Belo Horizonte performed data cleaning and consistency check, and it georeferenced each case to a census tract using the coordinates of the patient’s residence. Population counts for the census tracts in 2010 were obtained from WorldPop (Sorichetta et al., 2015).

### 2.3 Exposure: vegetation greenness

Our exposure variable is vegetation greenness derived from NDVI. NDVI represents the combined effect of vegetation quantity, including coverage and biomass, and quality, capturing photosynthetic activities and vegetation health (Tucker, 1979). NDVI takes values between – 1 and 1, with higher values indicating the larger quantity, better quality of vegetation, or both.

We calculated vegetation greenness as the census-tract average of annual mean of monthly maximum NDVI values. We first computed NDVI every 16 days in 2010 from the red and near-infrared electromagnetic bands (630–690 nm and 760–900 nm, respectively) of the Landsat-5 TM satellite surface reflectance images. Landsat-5 TM images have 30 m spatial resolution and 16-day temporal frequency. We masked out image areas covered by cloud, shadow and water, which would otherwise cause bias in NDVI and greenness estimates. Then for each month, we generated a monthly maximum NDVI image reflecting the greenest condition of each image pixel and averaged the monthly images to produce an NDVI image for 2010. We calculated the census-tract average of this 2010 NDVI image, which we subsequently used as vegetation greenness in our analyses. While, by definition, NDVI ranges between –1 and 1, for better interpretation of the model results, we rescaled greenness to a range between -10 and 10 (Fig. 1c). We accessed Landsat-5 data and performed these calculations in Google Earth Engine, a cloud-based platform for remote sensing image processing (Gorelick, 2013).

Because NDVI measures vegetation quantity and quality simultaneously, a census tract with healthy green vegetation that occupies a small proportion of its area might have a similar greenness value to a fully vegetated census tract with stressed or senescent and therefore less green vegetation. To better estimate the effect of vegetation quality on dengue incidence, we introduced additional covariates controlling for vegetation quantity within a census tract. These covariates include the percentage of census tract covered by forest, non-forest natural land, farmland, and building footprints. These covariates, along with others, are introduced below.

### 2.4 Other covariates

To reduce potential bias in the estimated association between dengue incidence and greenness, we controlled for a comprehensive set of covariates including socioeconomic vulnerability, population density, building height, the percentage of census tract area covered by building footprints, land cover composition, elevation, weather patterns, and neighborhood random effects. We identified these covariates initially using a directed acyclic graph (DAG) (Fig. S2), and we narrowed them down to a final set by examining multicollinearity with variance inflation factor (VIF). We collected these covariates from the year 2010 when possible to match the timeframe of the outcome and exposure variables. We used the Health Vulnerability Index (HVI) to measure socioeconomic vulnerability. This composite index, used by the City Health Department (Secretariat, 2018), is based on the 2010 census and encompasses infrastructure indicators and the socioeconomic status of residents (Fig. 1d). HVI considers infrastructure conditions, including water supply, sewage connection, and garbage collection, and it measures socioeconomic status by literacy rate, poverty, income, and percentage of the population in different racial groups. HVI is on a scale from 0 to 10, with higher values indicating more vulnerable areas. HVI influences both greenness and dengue incidence: vulnerable areas are often less resourceful to build and manage green space (Casey, James, Cushing, Jesdale, & Morello-Frosch, 2017); These areas also have undesirable water and sanitation infrastructures, which are suitable for the reproduction of *Aedes aegypti*.

We included population density, building height, the percentage of census tract covered by building footprints (i.e. building density), and land cover composition to further characterize the urban environment. These factors could be modified by urban planning and affect vegetation greenness in several ways. Areas with dense populations and buildings may have less open space for vegetation, leading to lower greenness. Land cover composition, including forest, non-forest natural land, farming, urban, water, and others, helps to separate the effect of vegetation quality from quantity on greenness magnitude. These urban environment characteristics also influence dengue incidence. Large building density and the presence of high-rise buildings are indicative of better housing conditions and less on-site water storage in the study area, which reduce breeding sites for *Aedes aegypti* (Seidahmed, Lu, Chong, Ng, & Eltahir, 2018; Troyo, Fuller, Calderón-Arguedas, Solano, & Beier, 2009). Land cover composition predetermines the suitability of *Aedes aegypti* habitats and the dynamics of the human population.

We calculated population density by dividing population counts from the 2010 WorldPop dataset (Sorichetta et al., 2015) by census tract area. We obtained height and footprint of individual buildings from BHGeo (https://bhgeo.pbh.gov.br/home). We retrieved 2010 land cover maps from the MapBiomass Brasil dataset (https://mapbiomas.org/en), a dataset that maps land cover annually with satellite remote sensing images at 30 m spatial resolution.

We also included land elevation and weather patterns to characterize the natural environment. These factors act as the background for vegetation development and influence *Aedes aegypti* population and dengue incidence. We approximated census-tract average elevation using the ALOS World 3D global digital surface model at 30 m resolution collected between 2006 and 2011 (Tadono et al., 2014). Although a digital surface model measures land elevation combined with the heights of above-ground objects (e.g., trees and buildings), this dataset was still valuable for this study as a proxy for land surface elevation, given the lack of spatial information on individual aboveground objects such as trees. For climate variables, we included census-tract averages of daily mean temperature, daily mean relative humidity, and annual total precipitation in 2010. We obtained annual total precipitation from the ERA5 dataset, which has 30 km spatial resolution and hourly temporal resolution (Copernicus Climate Change Service (C3S), 2017). We first generated annual total precipitation for each ERA5 grid cell by summing all hourly values in 2010. We then calculated census-tract precipitation as the area-weighted mean of the values from the ERA5 grid cells intersecting a census tract. We used the same procedure to calculate census-tract averages of daily mean temperature and daily mean relative humidity from the ERA5Land dataset (Copernicus Climate Change Service (C3S), 2019), which had a 9 km spatial resolution. It is worth noting that both ERA5 and ERA5Land are at coarse scales when compared with the census tracts.

After examining multicollinearity using VIF, we excluded several covariates of land cover composition, including % urban, % water, and % others from the model. Our final covariates and their summary statistics are in Table 1. The final covariates have a Spearman’s correlation between –0.64 and 0.37 with the exposure variable, greenness (Fig. S1). Furthermore, the covariates show low levels of multicollinearity, as their VIFs were all below 5 (Table 2), which is a commonly used empirical threshold for detecting multicollinearity (O’brien, 2007). In addition, the final covariates are confounders to the dengue-greenness relationship, which reduce bias in our estimation even when high multicollinearity between the exposure and these covariates exists. Multicollinearity does lead to wider confidence intervals, but confounding represents a greater concern in studies like ours (Schisterman, Perkins, Mumford, Ahrens, & Mitchell, 2017).

### 2.5 Regression model

We constructed four negative binomial mixed-effects models to estimate the association between dengue incidence and greenness. The mixed-effects model contains fixed effects that are the same across all census tracts and random effects that vary by neighborhood. The mixed-effects model makes use of our nested data structure (i.e., census tracts in neighborhoods, Fig. 1a), reduces omitted variable bias, and allows regression coefficients to vary by neighborhoods.

The first model included vegetation greenness with no covariates other than a neighborhood random intercept, *β_0j_* (equation (1)). Random intercept allows neighborhoods to have different baseline-level dengue incidence caused by unmeasured neighborhood characteristics such as vegetation species, management practices, and biophysical and built environments.

In the remaining models, we included additional covariates for socioeconomic vulnerability, population density, building height, percentage of census tract area covered by building footprints, land cover composition (% forest, % non-forest natural land, % farmland), elevation, and weather patterns (equation (2)). We further added a neighborhood random slope, *β_1j_*, for vegetation greenness to allow its coefficient vary by neighborhood (equation (3)). In this model, we allowed the random intercept, *β_0j_*, and random slope, *β_1j_*, to be correlated. Finally, we included an interaction term between vegetation greenness and socioeconomic vulnerability to test whether the association between dengue incidence and greenness was modified by socioeconomic vulnerability (equation (4)).

(1) $$\log(IR_{ij})=\beta_{0}+\beta_{1}{\text{greenness}}_{ij}+\beta_{0j}+\varepsilon_{ij}$$

(2) $$\log(IR_{ij})=\beta_{0}+\beta_{1}{\text{greenness}}_{ij}+\beta_{2}{\text{vulnerability}}_{ij}+\beta_{3}PD_{ij}+\beta_{4}{\text{buildingheight}}_{ij}+\beta_{5}{\text{buildingfootprint}}_{ij}+{\text{landcover}}_{ij}\delta +\beta_{6}{\text{elevation}}_{ij}+{\text{weather}}_{ij}\gamma +\beta_{0j}+\varepsilon_{ij}$$

(3) $$\log(IR_{ij})=\beta_{0}+\beta_{1}{\text{greenness}}_{ij}+\beta_{2}{\text{vulnerability}}_{ij}+\beta_{3}PD_{ij}+\beta_{4}{\text{buildingheight}}_{ij}+\beta_{5}{\text{buildingfootprint}}_{ij}+{\text{landcover}}_{ij}\delta +\beta_{6}{\text{elevation}}_{ij}+{\text{weather}}_{ij}\gamma +\beta_{0j}+\beta_{1j}{\text{greenness}}_{ij}+\varepsilon_{ij}$$

(4) $$\log(IR_{ij})=\beta_{0}+\beta_{1}{\text{greenness}}_{ij}+\beta_{2}{\text{vulnerability}}_{ij}+\alpha {\text{greenness}}_{ij}\times {\text{vulnerability}}_{ij}+\beta_{3}PD_{ij}+\beta_{4}{\text{buildingheight}}_{ij}+\beta_{5}{\text{buildingfootprint}}_{ij}+{\text{landcover}}_{ij}\delta +\beta_{6}{\text{elevation}}_{ij}+{\text{weather}}_{ij}\gamma +\beta_{0j}+\varepsilon_{ij}$$

where: *IR_ij_* is the incidence rate of dengue of census tract *i* in neighborhood *j*; *greenness_ij_* is census-tract average of annual mean of monthly maximum NDVI; *vulnerability_ij_* is Health Vulnerability Index measuring socioeconomic vulnerability; *PD_ij_* is population density; *buildingheight_ij_* is average building height; *buildingfootprint_ij_* is percentage of census tract area covered by buildings; *landcover_ij_* is a vector for land cover composition including the percentage of census area that is either forest, nonforest natural land, or farming; *elevation_ij_* is average land elevation in a census tract; *weather_ij_* is a vector of census-tract average of daily mean temperature, daily mean relative humidity, and annual total precipitation; *β_0j_* is the neighborhood random intercept; *β_1j_* is the neighborhood random slope for *greenness_ij_; ε_ij_* is a random error.

We used incidence rate ratios (IRRs) to interpret the model results. IRR is a factor by which the outcome, dengue cases per 100,000 population, changes with a one-unit change in a covariate, when holding all other covariates constant. Therefore, IRRs greater than 1 indicates a positive association, whereas IRRs less than 1 indicate a negative association.

In addition, to account for some of the spatial autocorrelations between the census tracts and heteroscedasticity of model residuals, we estimated neighborhood-cluster-robust standard errors for the model coefficients. The neighborhood-cluster-robust standard error assumes that census tracts within the same neighborhood (cluster) are correlated, but those between different neighborhoods are independent. This assumption is likely to result in larger standard errors, wider confidence intervals, and more statistically insignificant (conservative) inference when compared with assuming the census tracts are independent of each other. In this way, we partially controlled for spatial autocorrelation in our dataset.

### 2.6 Sensitivity tests

In addition to the main models (Eqs. (2)–(4)) using a mixed-effects negative binomial model with cluster-robust standard errors, we tested several alternatives to justify our choice. We started with a pooled model without neighborhood random intercepts, testing whether omitting such effects could bias the dengue-greenness relationship. We then estimated the models assuming the census tracts were independent of each other and model residuals were independently and identically distributed to illustrate how cluster-robust standard errors correct the statistical inference.

Finally, instead of a neighborhood random intercept model (equation (2)), we estimated a neighborhood fixed effects model. Both random intercept and fixed effects models are ways to control unobserved, group-level (neighborhood in this study) effects in hierarchical datasets by estimating a set of intercepts for the groups. The two models require different assumptions. A random intercept model assumes that these intercepts are uncorrelated with the covariates, whereas the fixed effects model relaxes such an assumption (Greene, 2018). While more versatile, we did not prioritize the fixed effects model as we would have a large number of fixed effects (n = 474, one per neighborhood), which leads to large confidence intervals for the coefficient estimates.

### 2.7 Limitations of the model

The first limitation of the study is that we used secondary data where cases were reported by health professionals, health services, and the public. Therefore, this case reporting was subject to misclassification bias. Dengue cases were reported to the Notifiable Diseases Information System, where cases could be misdiagnosed (de Mattos Almeida et al., 2007; Harris et al., 2000). In Belo Horizonte between 1996 and 2017, the surveillance system could confirm 46% of reported cases through laboratory diagnosis, whereas the remaining cases were diagnosed based on epidemiological criteria (Campos et al., 2019). Recent studies have shown improved accuracy in dengue case reporting in the Notifiable Diseases Information System (Barbosa, Barrado, Zara, & Siqueira, 2015; Goto et al., 2016)

Secondly, although we controlled for a comprehensive set of covariates, additional factors may further reduce omitted variable bias. These factors include vegetation species, land use, and irrigation practices, which affect both dengue incidence and greenness. However, quality datasets for these covariates were lacking when we conducted this study.

Thirdly, the environmental datasets in this study are at coarse spatial resolutions, which likely contain measurement errors and fail to capture fine-scale variations. For example, the 30 m resolution MapBiomass Brasil land cover product can only identify the main land cover, and classification accuracy is reduced in heterogeneous landscapes like wetlands due to the ‘mixed pixel’ problem (Kaur et al., 2019). This imprecise estimation of vegetation quantity prevents us from fully isolating the effect of vegetation quantity on greenness. The coarse spatial resolution also means a lack of within-neighborhood variation of the environmental variables to produce precise regression estimates. We consider that this issue exists in NDVI (30 m resolution) and land cover (30 m resolution), and it magnifies in weather pattern variables (9–31 km resolutions). Therefore, collecting fine-resolution environmental datasets is highly beneficial for environmental health studies to capture detailed variations in heterogeneous urban landscapes, reducing measurement error, and producing statistically sound results.

## 3 Results

### 3.1 Insignificant protective effect of greenness on dengue

We found a negative and statistically significant association (IRR < 1) between greenness and dengue incidence, when controlling only for neighborhood random effects (model 1, Table 3). In this model, a one-unit increase in greenness (40% of the mean of 2.52) was associated with a 16.6% ((0.834 – 1) × 100% = –16.6%, similar calculations hereafter) reduction in dengue incidence rate.

The negative association between greenness and dengue incidence attenuated and was not statistically significant after controlling for a set of covariates including socioeconomic vulnerability, population density, building height, percentage of census tract area covered by building footprints, elevation, land cover composition, and weather patterns (model 2, Table 3). In the latter model, a one-unit increase in greenness was associated with a 5.1% reduction in dengue incidence.

### 3.2 Socioeconomic vulnerability modifies the dengue-greenness association

We observed a statistically significant random slope for greenness, indicating the association between greenness on dengue incidence varied by neighborhood (model 3, Table 3). In addition, this model estimated a stronger and statistically significant protective effect of greenness, where a one-unit increase in greenness reduced dengue incidence by 8.5%.

**Fig. 2:**
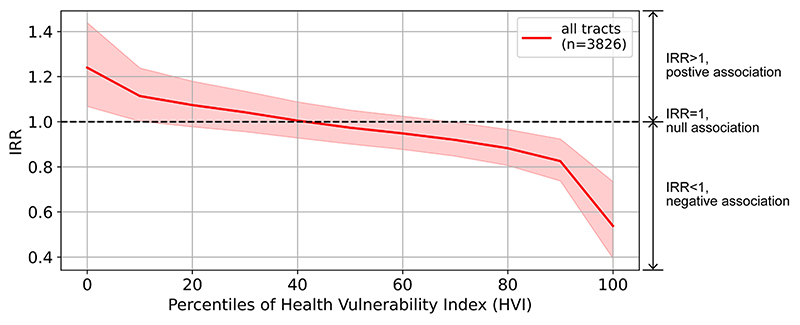
Associations (IRR) between dengue incidence and vegetation greenness, conditioned on socioeconomic vulnerability.. IRR is the factor by which dengue incidence (annual dengue cases per 100,000 residents) changes for a one-unit increase in a covariate, when holding other covariates constant. Socioeconomic vulnerability is measured by the Health Vulnerability Index. 95% confidence intervals of the IRRs are shaded.

We observed a statistically significant negative interaction between greenness and socioeconomic vulnerability (model 4, Table 3), suggesting that the association between dengue incidence and greenness was modified by socioeconomic vulnerability. The association between dengue incidence and greenness shifted from positive (IRR > 1) to negative (IRR < 1) as the census tracts in our sample became more vulnerable (Fig. 2). This association was stronger and statistically significant in the census tracts with low (roughly below the 10th percentile) or high (roughly above the 70th percentile) HVI.

### 3.3 The associations between dengue incidence and the covariates

We found that socioeconomic vulnerability, % building footprint and % forest had positive and statistically significant associations with dengue incidence across the models (models 2–4, Table 3). In addition, % non-forest natural land and total precipitation had positive but statistically insignificant associations with dengue incidence.

We found negative and statistically significant associations between dengue incidence and population density, % farmland, average elevation, relative humidity, and temperature (models 2–4, Table 3). We found negative but statistically insignificant association between dengue incidence and average building height.

### 3.4 Sensitivity tests

The alternative model specifications (Table S1) generally confirmed the dengue-greenness relationship in our main models (Table 3). However, the sizes of the coefficients and their confidence intervals varied due to different model assumptions. Compared with the main models, the pooled model ignoring neighborhood effects (model 2–1, Table S1) produced a stronger negative and statistically significant association (IRR = 0.812) between dengue incidence and greenness. However, this association was likely biased due to omitted variables. Specifying cluster-robust standard errors led to wider confidence intervals and more conservative statistical inference, when compared with using independent standard errors (models 2–2, 3–1, 4–1, Table S1). Finally, the neighborhood fixed effects model yielded a slightly weaker association between dengue and greenness (model 2–3, Table S1).

## 4 Discussion

### 4.1 Protective effect of vegetation greenness on dengue incidence

Our results suggested a negative but statistically not significant association between dengue incidence and vegetation greenness averaged across the census tracts in Belo Horizonte, after controlling for socioeconomic vulnerability, population density, building height, percentage of census tract area covered by building footprints, elevation, land cover composition, weather patterns, and neighborhood random intercept (model 2, Table 3). Furthermore, this association varied by neighborhood (model 3, Table 3). For example, it changed based on the socioeconomic vulnerability of the census tracts: the association was negative and statistically significant for the most vulnerable census tracts, but it was positive and statistically significant for the least vulnerable ones (Fig. 2).

Greenness, as measured by NDVI, alone represents the combined effect of vegetation quantity and quality. We attempted to separate the effect of vegetation quantity from quality by introducing covariates for vegetation coverage, including land cover composition and the percentages of census tract area covered by buildings. Despite some data limitations discussed in Section 2.7, higher greenness likely indicated better vegetation quality in our analyses.

Our results align in part with previous findings indicating a negative association between dengue incidence and vegetation regarding quantity (Araujo et al., 2015; Cao et al., 2017) and the combined effect of quantity and quality (Meza-Ballesta & Gónima, 2014). However, other studies have also reported positive (Martínez-Bello et al., 2017) or non-linear associations (Qi et al., 2015). We suspect that the inconsistencies reflect differences in spatial scales of analysis, measurements of vegetation, and analytical approaches. For instance, some studies only analyzed simple correlations between dengue incidence and vegetation without controlling for a comprehensive set of covariates as in our study (Cao et al., 2017; Martínez-Bello et al., 2017). It is also worth noting that the overall negative association between dengue incidence and greenness identified in our study was small and not statistically significant. One explanation could be that the main factor leading to high *Aedes aegypti* population (the main regional dengue vector) and consequently high dengue incidence include household water storage, sewage connection, and trash collection, rather than vegetation itself. It may also be the case that the log-linear relationship assumed by negative binomial models is an oversimplification, as threshold effect (“tipping points”) may exist in the actual dengue-greenness relationship. To account for the threshold effect, researchers have employed higher-order terms of independent variables (Spencer, 2013), semi-parametric models (Ding, Cao, Yu, & Ju, 2021), and machine learning models (Zhao, Yan, Yu, & Van Hentenryck, 2020).

To our knowledge, no previous studies have examined whether the association between dengue incidence and greenness is modified by socioeconomic vulnerability. Our results (model 4, Table 3) show that while census tracts with lower socioeconomic vulnerability had a positive association between dengue incidence and greenness, the association was negative in census tracts with higher socioeconomic vulnerability (Fig. 2). This pattern may indicate that other vegetation characteristics, such as vegetation species, type (natural versus cultivated, tree versus grass), structure, and management practices differ between census tracts with different levels of socioeconomic vulnerability (Mitchell et al., 2016). For example, in Belo Horizonte, many vulnerable census tracts are close to the city border (Fig. 1d), which is abundant in natural vegetation not suitable for *Aedes aegypti* (Dorvillé, 2010; Medeiros-Sousa et al., 2017). The same one-unit increase in the greenness of different vegetation could lead to variable changes in dengue incidence. This also may explain why prior studies have found mixed relationships between dengue incidence and vegetation, as vegetation can be systematically different between their study areas.

### 4.2 Benefits of improved built environment and housing

Our models also estimated the associations between dengue incidence and additional covariates of socioeconomic vulnerability, urban and natural environment. The disease process is complex, and particular microenvironments can become “pathogenic landscape” (Marti et al., 2020). While the socioeconomic vulnerability, urban, and natural environment covariates studied here are markers of a “pathogenic landscape” and may lead to interventions, any causality should be carefully interpreted from our results due to omitted variable bias and study design issues (Spencer et al., 2020). We focus our discussion here on socioeconomic vulnerability and urban environment covariates, as they could be improved by social policy and urban planning.

The positive association between dengue incidence and socioeconomic vulnerability (HVI) found in this study (models 2–3, Table 3) is consistent with previous ones in this urban setting (Campos et al., 2019; de Mattos Almeida et al., 2007). We consider this positive association highly likely, as supported by the construct of HVI and vector biology. High HVI indicates inadequate water supply infrastructure, sewage connection, and garbage collection, creating favorable conditions for *Aedes aegypti* infestation (Gubler, 2011).

Census tract covered by high fractions of building footprint (i.e., building density) and forest showed positive associations with dengue incidence. High building density may be associated with extensive drainage networks (Seidahmed et al., 2018), and forest provides shading (Barrera et al., 2006) – both are favorable conditions for *Aedes aegypti.* Population density and % farmland showed a negative association with dengue incidence. While increased population density means higher chances of transmission, it may also indicate a better supply of water infrastructures and possibilities of herd immunity. The association between % farmland and dengue incidence is likely dependent on agriculture practices and requires further investigation in our study area.

### 4.3 Urban planning and policy implications

Local governments should consider vegetation management strategies to improve vegetation quality, particularly in vulnerable areas with greater potential benefits. Preserving green space has been included in Belo Horizonte’s development agendas, such as the Organic Law of the Municipality, the municipal conferences on urban policies in 2014, and the city’s master plan in 2019 (Costa, Álvares, Maciel, Teixeira, Coimbra, & de Simão, 2009; Cruz, 2020). When preserving green space, we suggest local governments restore typical, native forest understory and arboreal landscapes. When well-managed, these landscapes could largely decrease the presence of vectors for dengue and other infectious diseases (Pedrosa et al., 2020). Most problems with green space in Brazilian cities, as happens in Belo Horizonte, are related to poor management, which leads to habitat degradation and trash accumulation (Cardoso, Vasconcellos Sobrinho, & de Vasconcellos, 2015; Lobo, 2020). These poorly managed areas are therefore more likely to become potential breeding sites for disease vectors. Managing vegetation not only reduces dengue risk but also brings co-benefits such as reducing crime, mitigating urban heat, simulating physical activities, and improving mental health (Cardoso et al., 2015; Casey et al., 2017; James, Banay, Hart, & Laden, 2015). Managing tree canopy to reduce shading, mainly when water bodies are present, may also be an option, although other benefits of trees should be considered.

Green space management should come together with improvements in water supply and drainage infrastructures, and sanitation services to reduce the likelihood of having a “pathogenic landscape”. Furthermore, the city may consider adopting an optimized drainage network to avoid an extensive length of pipes, which further reduces *Aedes aegypti* habitat. One priority area for these interventions is informal settlements in Belo Horizonte. The city began to improve the living conditions of informal settlements in 1970, and its recent efforts have been led by the Vila Viva Project since 2005 (Friche, Dias, Reis, Dias, & Caiaffa, 2015). This project creates the opportunity for future studies to quantify how bundled interventions, including improvements in sanitation and housing conditions, and provision of green space, together change dengue risk.

## 5 Conclusions

We found an overall negative but statistically not significant association between dengue and greenness across 3826 census tracts in Belo Horizonte, Brazil, after controlling for a comprehensive set of covariates. We also found that the dengue-greenness association was modified by socioeconomic vulnerability: the association was positive in the least vulnerable census tracts but negative in the most vulnerable areas. Since we controlled for vegetation quantity using land cover composition and percentage of census tract area covered by building footprints, high greenness here likely indicates higher vegetation quality in terms of better vegetation health, more vigorous photosynthetic activities, and less environmental degradation.

Collectively, our results show the potential role of improved vegetation quality, as manifested by higher greenness, in reducing dengue risk, particularly for vulnerable areas. When well-managed, vegetated areas are less likely to become breeding sites for dengue vectors and reduce the risk of local dengue transmission. While we showed the potential role of improving vegetation quality in reducing dengue risk, this strategy should be incorporated with other multisectoral approaches, such as improvements of water supply and drainage infrastructures, to reduce the presence of “pathogenic landscape” and dengue risk.

## Supplementary Material

Supplementary file 1

## Figures and Tables

**Table 1:** Summary statistics of outcome variable, exposure variable, and covariates.

	Max.	Min.	Mean	Median	25th percentile	75th percentile	Std.	Std., between neighborhood	Std., within the neighborhood
Incidence rate (cases/100,000 population)	6756.48	0.00	229.18	138.11	46.03	306.00	307.52	447.49	186.09
Greenness (-10-10)	6.69	0.93	2.52	2.39	2.07	2.79	0.71	0.72	0.50
Vulnerability (HVI, 0-10)	6.96	0.47	2.41	2.35	1.70	2.93	0.92	1.06	0.38
Population density (people/ hectare)	8476.80	0.78	1306.15	1110.57	818.82	1578.70	808.10	701.56	571.25
Average building height (m)	96.25	2.43	7.11	5.24	4.74	6.65	5.49	3.15	3.27
% building footprint	80.92	0.19	40.59	42.72	34.53	48.13	12.11	12.03	8.70
% forest	68.60	0.00	0.60	0.00	0.00	0.00	4.29	3.93	3.61
% non-forest natural land	31.65	0.00	0.03	0.00	0.00	0.00	0.75	0.53	0.65
% farmland	71.09	0.00	1.06	0.00	0.00	0.00	5.75	6.76	4.30
Average elevation (m)	1235.49	697.23	876.52	867.51	817.75	924.97	74.95	82.63	19.01
Relative humidity (%)	73.59	70.14	71.35	71.54	71.11	71.90	0.76	0.73	0.16
Total precipitation (mm/year)	100.36	83.47	90.93	94.33	83.47	94.33	5.02	5.12	0.77
Temperature (°C)	21.22	19.44	20.43	20.55	20.41	20.55	0.49	0.52	0.10
Census tract area (hectare)	1288.10	0.08	7.95	4.98	2.61	7.87	27.82	35.15	19.63
Total population (people)	24,298	32	5889	5869	3801	7887	2999.81	2457.93	2611.48

Number of census tracts = 3826,

Number neighborhoods = 474, each containing between 1 and 58 census tracts

**Table 2:** Variable inflation factor (VIF) for the exposure variable and covariates.

	VIF
Greenness (-10–10)	2.88
Vulnerability (HVI, 0–10)	1.64
Population density (people/hectare)	1.65
Average building height (m)	1.61
% building footprint	2.41
% forest	1.51
% non-forest natural land	1.07
% farmland	1.71
Average elevation (m)	3.33
Relative humidity (%)	2.60
Total precipitation (mm/year)	2.48
Temperature (° C)	3.74

**Table 3:** Results of models examining the association between dengue incidence and greenness, controlling for socioeconomic and environmental covariates.

	(1)IRR	(2)IRR	(3)IRR	(4)IRR
*Fix effects* Greenness (-10–10)	0.834^***^ [0.789,0.882]	0.949 [0.878,1.025]	0.915^**^ [0.843,0.994]	1.316^***^ [1.108,1.564]
Vulnerability (HVI, 0–10)		1.254^***^ [1.162,1.354]	1.269^***^ [1.176,1.368]	1.824^***^ [1.509,2.205]
Greenness × Vulnerability				0.879^***^ [0.825,0.938]
Population density (people/hectare)		0.9998^***^ [0.9997,0.9998]	0.9998^***^ [0.9997,0.9998]	0.9997^***^ [0.9997,0.9998]
Average building height (m)		0.982 [0.960,1.005]	0.980 [0.956,1.005]	0.985 [0.963,1.007]
% building footprint		1.017^***^ [1.013,1.022]	1.017^***^ [1.013,1.022]	1.017^***^ [1.013,1.022]
% forest		1.007* [1.000,1.015]	1.007* [0.999,1.016]	1.005 [0.998,1.012]
% non-forest natural land		1.017 [0.980,1.055]	1.020 [0.983,1.058]	1.010 [0.976,1.045]
% farmland		0.987^***^ [0.980,0.995]	0.988^***^ [0.981,0.995]	0.992^**^ [0.984,0.999]
Average elevation (m)		0.993^***^ [0.992,0.995]	0.994^***^ [0.992,0.995]	0.993^***^ [0.992,0.994]
Relative humidity (%)		0.707^***^ [0.605,0.827]	0.699^***^ [0.598,0.817]	0.700^***^ [0.600,0.816]
Total precipitation (mm/year)		1.012 [0.997,1.027]	1.010 [0.995,1.025]	1.016^**^ [1.000,1.032]
Temperature (°C)		0.775* [0.593,1.013]	0.784* [0.601,1.023]	0.775* [0.596,1.008]
*Random effects* ^+^ var(Greenness)			0.050^***^ [0.022,0.115]	
var(Intercept)	1.101^***^ [0.952,1.272]	0.560^***^ [0.461,0.680]	0.689^***^ [0.420,1.129]	0.532^***^ [0.440,0.644]
Controls	No	Yes	Yes	Yes
Random intercept	Yes	Yes	Yes	Yes
Random slope	No	No	Yes	No
Confidence interval	Neighborhood-cluster-robust	Neighborhood-cluster-robust	Neighborhood-cluster-robust	Neighborhood-cluster-robust
Number of observations	3826	3826	3826	3826

Note: A coefficient, or incidence rate ratio (IRR), is the factor by which the dengue incidence (dengue cases per 100,000 residents) changes for a one-unit increase in the corresponding covariate when holding other covariates constant. Vulnerability is measured by the Health Vulnerability Index (HVI) that encompasses infrastructure indicators related to basic sanitation and socioeconomic status of residents, recorded by the 2010 census.

*, **, and *** indicate significant at p-value < 0.10, p-value < 0.05, and p-value < 0.01. 95% confidence intervals are in square brackets. +Random effects are the variances of log-transformed coefficients (IRRs).

## Highlights

- Vegetation greenness is negatively but insignificantly associated with dengue incidence.
- This association is stronger in socioeconomically vulnerable areas.
- Improved vegetation quality has the potential to reduce dengue risk.
- Openly accessible, coarse resolution environmental data have limitations in environmental health studies.

## References

[R1] de Almeida MCM, Assunção RM, Proietti FA, Caiaffa WT (2008). Dinâmica intra-urbana das epidemias de dengue em Belo Horizonte, Minas Gerais, Brasil, 1996-2002. Cadernos de Saúde Pública.

[R2] Araujo RV, Albertini MR, Costa-da-Silva AL, Suesdek L, Franceschi NCS, Bastos NM, Katz G, Cardoso VA, Castro BC, Capurro ML, Allegro VLAC (2015). São Paulo urban heat islands have a higher incidence of dengue than other urban areas. The Brazilian Journal of Infectious Diseases.

[R3] de Bairros BH (2009). What is a neighborhood? : Belo Horizonte neighborhoods.

[R4] Barbosa JR, dos S Barrado JC, de S Zara Amâncio AL, Siqueira João B (2015). Avaliação da qualidade dos dados, valor preditivo positivo, oportunidade e representatividade do sistema de vigilância epidemiológica da dengue no Brasil, 2005 a 2009. Epidemiologia e Serviços de Saúde.

[R5] Barrera R, Amador M, Clark GG (2006). Use of the pupal survey technique for measuring Aedes aegypti (Diptera: Culicidae) productivity in Puerto Rico. The American Journal of Tropical Medicine and Hygiene.

[R6] Bavia L, Melanda FN, de Arruda TB, Mosimann ALP, Silveira GF, Aoki MN, Bordignon J (2020). Epidemiological study on dengue in southern Brazil under the perspective of climate and poverty. Scientific Reports.

[R7] Belo Horizonte (2016). VULNERABILITY ASSESSMENT TO CLIMATE CHANGE. IN THE MUNICIPALITY OF BELO HORIZONTE - BRAZIL. SUMMARY FOR POLICYMAKERS.

[R8] Belo Horizonte (2020). Dengue. Prefeitura de Belo Horizonte. http://prefeitura.pbh.gov.br/saude/informacoes/vigilancia/vigilancia-epidemiologica/doencas-transmissiveis/dengue.

[R9] Bhatt S, Gething PW, Brady OJ, Messina JP, Farlow AW, Moyes CL, Hay SI (2013). The global distribution and burden of dengue. Nature.

[R10] Bona ACD, Twerdochlib AL, Navarro-Silva MA (2011). Detecção do vírus da dengue em populaçães naturais de mosquitos. Boletín de Malariología y Salud Ambiental.

[R11] Bueno M do CD, D’Antona A de O (2017). A GEOGRAFIA DO CENSO NO BRASIL: POTENCIALIDADES E LIMITAÇOES NA EXECUÇÃO DE ANÃLISES ESPACIAIS. Geographia Niterói.

[R12] Buonsenso D, Barone G, Onesimo R, Calzedda R, Chiaretti A, Valentini P (2014). The re-emergence of dengue virus in non-endemic countries: A case series. BMC Research Notes.

[R13] Bustamante MMC, Metzger JP, Scariot AO, Bager A, Turra A, Barbieri A, Neves A, Boesing AL, Agostinho AA, Marques AC, Dias B (2019). Tendências e impactos dos vetores de degradação e restauração da biodiversidade e dos serviços ecossistêmicos.

[R14] Campos NBD, Morais MHF, Ceolin APR, Cunha M da CM, Nicolino RR, Schultes OL, Friche AAdeL, Caiaffa WT (2019). Twenty-Two years of dengue fever (1996-2017): An epidemiological study in a Brazilian city. International Journal of Environmental Health Research.

[R15] Cao Z, Liu T, Li X, Wang J, Lin H, Chen L, Wu Z, Ma W (2017). Individual and interactive effects of socio-ecological factors on dengue fever at fine spatial scale: A geographical detector-based analysis. International Journal of Environmental Research and Public Health.

[R16] Cardoso SLC, Vasconcellos Sobrinho M, Vasconcellos AMA, Cardoso SLC, Vasconcellos Sobrinho M, Vasconcellos AMdeA (2015). Gestaão ambiental de parques urbanos: O caso do Parque Ecolégico do Município de Belém Gunnar Vingren. urbe Revista Brasileira de Gestaão Urbana.

[R17] Casey J, James P, Cushing L, Jesdale B, Morello-Frosch R (2017). Race, Ethnicity, Income Concentration and 10-Year Change in Urban Greenness in the United States. International Journal of Environmental Research and Public Health.

[R18] Colwell R (1998). Balancing the biocomplexity of the planet’s living systems: A twenty-first century task for science. BioScience.

[R19] Copernicus Climate Change Service (C3S) (2017). ERA5: Fifth generation of ECMWF atmospheric reanalyses of the global climate. Copernicus Climate Change Service Climate Data Store (CDS).

[R20] Copernicus Climate Change Service (C3S) (2019). ERA5-Land hourly data from 2001 to present.

[R21] Cordeiro R, Donalisio MR, Andrade VR, Mafra AC, Nucci LB, Brown JC, Stephan C (2011). Spatial distribution of the risk of dengue fever in southeast Brazil, 2006–2007. BMC Public Health.

[R22] Costa SAP, Álvares LC, Maciel MC, Teixeira MCV, Coimbra VBC, Simaão KMdeC, Perna S de A, Godinho LR (2009). Os Espaços Livres na Paisagem de Belo Horizonte. Paisagem e Ambiente.

[R23] Cruz MM (2020). Plano Diretor de BH entra em vigor com meta de garantir sustentabilidade. Estado de Minas.

[R24] de Almeida AS, Medronho RdeA, Valencia LIO (2009). Spatial analysis of dengue and the socioeconomic context of the city of Rio de Janeiro (Southeastern Brazil). Revista De Saude Publica.

[R25] de Macêdo SF, Silva KA, de Vasconcelos RB, de Sousa IV, Mesquita LPS, Barakat RDM, de Oliveira Lima JW (2021). Scaling up of Eco-Bio-Social Strategy to Control Aedes aegypti in Highly Vulnerable Areas in Fortaleza, Brazil: A Cluster, Non-Randomized Controlled Trial Protocol. International Journal of Environmental Research and Public Health.

[R26] de Mattos Almeida MC, Caiaffa WT, Assunçãao RM, Proietti FA (2007). Spatial Vulnerability to Dengue in a Brazilian Urban Area During a 7-Year Surveillance. Journal of Urban Health.

[R27] Degallier N, Teixeira José M Soécrates, Soares S da S, Pereira RD, Pinto SCF, Chaib AdeJM, Oliveira E (2003). Aedes albopictus may not be vector of dengue virus in human epidemics in Brazil. Revista de Saúde Pública.

[R28] Degroote S, Zinszer K, Ridde V (2018). Interventions for vector-borne diseases focused on housing and hygiene in urban areas: A scoping review. Infectious Diseases of Poverty.

[R29] Ding C, Cao X, Yu B, Ju Y (2021). Non-linear associations between zonal built environment attributes and transit commuting mode choice accounting for spatial heterogeneity. Transportation Research Part A: Policy and Practice.

[R30] Donalisio MR, Freitas ARR, Zuben APBV, Donalisio MR, Freitas ARR, Zuben APBV (2017). Arboviruses emerging in Brazil: Challenges for clinic and implications for public health. Revista de Saúde Pública.

[R31] Dorvillé LF (2010). Mosquitoes as Bioindicators of Forest Degradation in Southeastern Brazil, a Statistical Evaluation of Published Data in the Literature. Studies on Neotropical Fauna and Environment.

[R32] Ellis BR, Wilcox BA (2009). The ecological dimensions of vector-borne disease research and control. Cadernos De Saude Publica.

[R33] de Friche AAL, de Dias MAS, dos Reis PB, Dias CS, Caiaffa WT, BH-Viva Project (2015). Urban upgrading and its impact on health: A “quasi-experimental” mixed-methods study protocol for the BH-Viva Project. Cadernos De Saude Publica.

[R34] Friche AAdeL, Dias MAdeS, Reis PB, dos Dias CS, Caiaffa WT (2015). Intervenções de requalificação urbana e o impacto na saúde: Protocolo de estudo “quasi-experimental” com métodos mistos - Projeto BH-Viva. Cadernos de Saúde Pública.

[R35] Gorelick N (2013). Google Earth Engine.

[R36] Goto DYN, Larocca LM, Felix JVC, Kobayashi VL, Chaves MMN, Goto DYN, Chaves MMN (2016). Avaliação da oportunidade de notificação da dengue no Estado do Paraná. Acta Paulista de Enfermagem.

[R37] Greene WH (2018). Econometric Analysis.

[R38] Gubler DJ (1998). Dengue and Dengue Hemorrhagic Fever. Clinical Microbiology Reviews.

[R39] Gubler DJ (2011). Dengue, Urbanization and Globalization: The Unholy Trinity of the 21st Century. Tropical Medicine and Health.

[R40] Harris E, Videa E, Pérez L, Sandoval E, Téllez Y, Péerez M, Balmaseda A (2000). Clinical, epidemiologic, and virologic features of dengue in the 1998 epidemic in Nicaragua. The American Journal of Tropical Medicine and Hygiene.

[R41] Higa Y (2011). Dengue vectors and their spatial distribution. Tropical Medicine and Health.

[R42] Honorato T, Lapa PPdeA, Sales CMM, Reis-Santos B, Tristaão-Saé R, Bertolde A Inéacio, Maciel ELN (2014). Spatial analysis of distribution of dengue cases in Espirito Santo, Brazil, in 2010: Use of Bayesian model. Revista Brasileira de Epidemiologia.

[R43] Huang C-C, Tam T, Chern Y-R, Lung S-C, Chen N-T, Wu C-D (2018). Spatial clustering of dengue fever incidence and its association with surrounding greenness. International Journal of Environmental Research and Public Health.

[R44] IBGE (2010). Censo de Belo Horizonte 2010.

[R45] James P, Banay RF, Hart JE, Laden F (2015). A review of the health benefits of greenness. Current Epidemiology Reports.

[R46] Jiannino JA, Walton WE (2004). Evaluation of vegetation management strategies for controlling mosquitoes in a southern California constructed wetland. Journal of the American Mosquito Control Association.

[R47] Kaur S, Bansal RK, Mittal M, Goyal LM, Kaur I, Verma A, Son LH (2019). Mixed pixel decomposition based on extended fuzzy clustering for single spectral value remote sensing images. Journal of the Indian Society of Remote Sensing.

[R48] Lee YS (1994). Urban planning and vector control in Southeast Asian cities. Gaoxiong Yi Xue Ke Xue Za Zhi = The Kaohsiung Journal of Medical Sciences.

[R49] Lindsay SW, Wilson A, Golding N, Scott TW, Takken W (2017). Improving the built environment in urban areas to control Aedes aegypti-borne diseases. Bulletin of the World Health Organization.

[R50] Lobo ME (2020). Prefeitura promove “‘Dengue Zero’” em 70 parques da capital.

[R51] Marti R, Li Z, Catry T, Roux E, Mangeas M, Handschumacher P, Gong P (2020). A mapping review on urban landscape factors of dengue retrieved from earth observation data, GIS techniques, and survey questionnaires. Remote Sensing.

[R52] Martínez-Bello DA, López-Quílez A, Torres Prieto A (2017). Relative risk estimation of dengue disease at small spatial scale. International Journal of Health Geographics.

[R53] Medeiros-Sousa AR, Fernandes A, Ceretti-Junior W, Wilke ABB, Marrelli MT (2017). Mosquitoes in urban green spaces: Using an island biogeographic approach to identify drivers of species richness and composition. Scientific Reports.

[R54] Meza-Ballesta A, Gónima L (2014). The influence of climate and vegetation cover on the occurrence of dengue cases (2001-2010).

[R55] Mitchell MGE, Wu D, Johansen K, Maron M, McAlpine C, Rhodes JR, Lee TM (2016). Landscape structure influences urban vegetation vertical structure. Journal of Applied Ecology.

[R56] Municipal Health Secretariat (2018). Índice de Vulnerabilidade da Saúde (IVS-BH). Prefeitura de Belo Horizonte.

[R57] O’brien RM (2007). A Caution Regarding Rules of Thumb for Variance Inflation Factors. Quality & Quantity.

[R58] Ogden N (2016). Vector-borne disease, climate change and urban design. Canada Communicable Disease Report.

[R59] Pedrosa MC, Borges MAZ, Eiras AE, Caldas S, Cecílio AB, Brito MF, Ribeiro SP (2020). Invasion of Tropical Montane Cities by Aedes aegypti and Aedes albopictus (Diptera: Culicidae) Depends on Continuous Warm Winters and Suitable Urban Biotopes. Journal of Medical Entomology.

[R60] Penso-Campos JM, Fraga E, Caldas E, Sommer JAP, Peérico E (2018). Aspectos da paisagem e fatores socioeconômicos nos casos de dengue na cidade de Porto Alegre, RS (Landscape aspects and socioeconomic factors in the cases of Dengue in the city of Porto Alegre, RS). Revista Brasileira de Geografia Física.

[R61] Pessanha JEM, Caiaffa WT, Kroon EG, Proietti FA (2010). Dengue em três distritos sanitários de Belo Horizonte, Brasil: Inquéerito soroepidemiológico de base populacional, 2006 a 2007. Revista Panamericana de Salud Pública.

[R62] Powell JR, Tabachnick WJ (2013). History of domestication and spread of Aedes aegypti—A Review. Memórias Do Instituto Oswaldo Cruz.

[R63] Qi X, Wang Y, Li Y, Meng Y, Chen Q, Ma J, Harley D (2015). The effects of socioeconomic and environmental factors on the incidence of dengue fever in the Pearl River Delta, China, 2013. PLoS Neglected Tropical Diseases.

[R64] Rojas-Rueda D, Nieuwenhuijsen MJ, Gascon M, Perez-Leon D, Mudu P (2019). Green spaces and mortality: A systematic review and meta-analysis of cohort studies. The Lancet Planetary Health.

[R65] Salje H, Lessler J, Endy TP, Curriero FC, Gibbons RV, Nisalak A, Cummings DAT (2012). Revealing the microscale spatial signature of dengue transmission and immunity in an urban population. Proceedings of the National Academy of Sciences of the United States of America.

[R66] San Martín JL, Brathwaite O, Zambrano B, Solórzano JO, Bouckenooghe A, Dayan GH, Guzmán MG (2010). The epidemiology of dengue in the americas over the last three decades: A worrisome reality. The American Journal of Tropical Medicine and Hygiene.

[R67] Sari SYI, Adelwin Y, Rinawan FR (2020). Land use changes and cluster identification of dengue hemorrhagic fever cases in Bandung, Indonesia. Tropical Medicine and Infectious Disease.

[R68] Schisterman EF, Perkins NJ, Mumford SL, Ahrens KA, Mitchell EM (2017). Collinearity and causal diagrams – a lesson on the importance of model specification. Epidemiology (Cambridge, Mass).

[R69] Seidahmed OME, Lu D, Chong CS, Ng LC, Eltahir EAB (2018). Patterns of Urban Housing Shape Dengue Distribution in Singapore at Neighborhood and Country Scales. GeoHealth.

[R70] Sorichetta A, Hornby GM, Stevens FR, Gaughan AE, Linard C, Tatem AJ (2015). High-resolution gridded population datasets for Latin America and the Caribbean in 2010, 2015, and 2020. Scientific Data.

[R71] Spencer JH (2013). The urban health transition hypothesis: Empirical Evidence of an Avian Influenza Kuznets Curve in Vietnam?. Journal of Urban Health.

[R72] Spencer JH, Finucane ML, Fox JM, Saksena S, Sultana N (2020). Emerging infectious disease, the household built environment characteristics, and urban planning: Evidence on avian influenza in Vietnam. Landscape and Urban Planning.

[R73] Tadono T, Ishida H, Oda F, Naito S, Minakawa K, Iwamoto H (2014). Precise Global DEM Generation by ALOS PRISM. ISPRS Annals of Photogrammetry, Remote Sensing and Spatial Information Sciences.

[R74] de Teixeira TRA, de Medronho RA (2008). Indicadores sócio-demográficos e a epidemia de dengue em 2002 no Estado do Rio de Janeiro, Brasil. Cadernos de Saúde Pública.

[R75] Thullen JS, Sartoris JJ, Walton WE (2002). Effects of vegetation management in constructed wetland treatment cells on water quality and mosquito production. Ecological Engineering.

[R76] Troyo A, Fuller DO, Calderón-Arguedas O, Solano ME, Beier JC (2009). Urban structure and dengue fever in Puntarenas, Costa Rica. Singapore Journal of Tropical Geography.

[R77] Tucker CJ (1979). Red and photographic infrared linear combinations for monitoring vegetation. Remote Sensing of Environment.

[R78] Vargas WP, Kawa H, Sabroza PC, Soares VB, Honório NA, de Almeida AS (2015). Association among house infestation index, dengue incidence, and sociodemographic indicators: Surveillance using geographic information system. BMC Public Health.

[R79] Wilcox BA, Colwell RR (2005). Emerging and reemerging infectious diseases: Biocomplexity as an interdisciplinary paradigm. EcoHealth.

[R80] Ximenes R, Amaku M, Lopez LF, Coutinho FAB, Burattini MN, Greenhalgh D, Massad E (2016). The risk of dengue for non-immune foreign visitors to the 2016 summer olympic games in Rio de Janeiro, Brazil. BMC Infectious Diseases.

[R81] Xu L, Stige LC, Chan K-S, Zhou J, Yang J, Sang S, Stenseth NC (2017). Climate variation drives dengue dynamics. Proceedings of the National Academy of Sciences.

[R82] Zhao X, Yan X, Yu A, Van Hentenryck P (2020). Prediction and behavioral analysis of travel mode choice: A comparison of machine learning and logit models. Travel Behaviour and Society.

[R83] Zhu G, Liu J, Tan Q, Shi B (2016). Inferring the Spatio-temporal Patterns of Dengue Transmission from Surveillance Data in Guangzhou, China. PLOS Neglected Tropical Diseases.

