# Supplementary material for: Disentangling associations between vegetation greenness and dengue in a Latin American city: Findings and challenges
Source: Landsc Urban Plan. Author manuscript; Available in PMC 2021 Dec 1. (PMC8519391; doi:10.1016/j.landurbplan.2021.104255)
Supplement: Supplementary file 1 [file EMS136002-supplement-Supplementary_file_1.pdf]

Supporting Information

Disentangling associations between vegetation greenness and dengue in a Latin American city: findings and challenges

Number of figures:2

Number of tables: 1

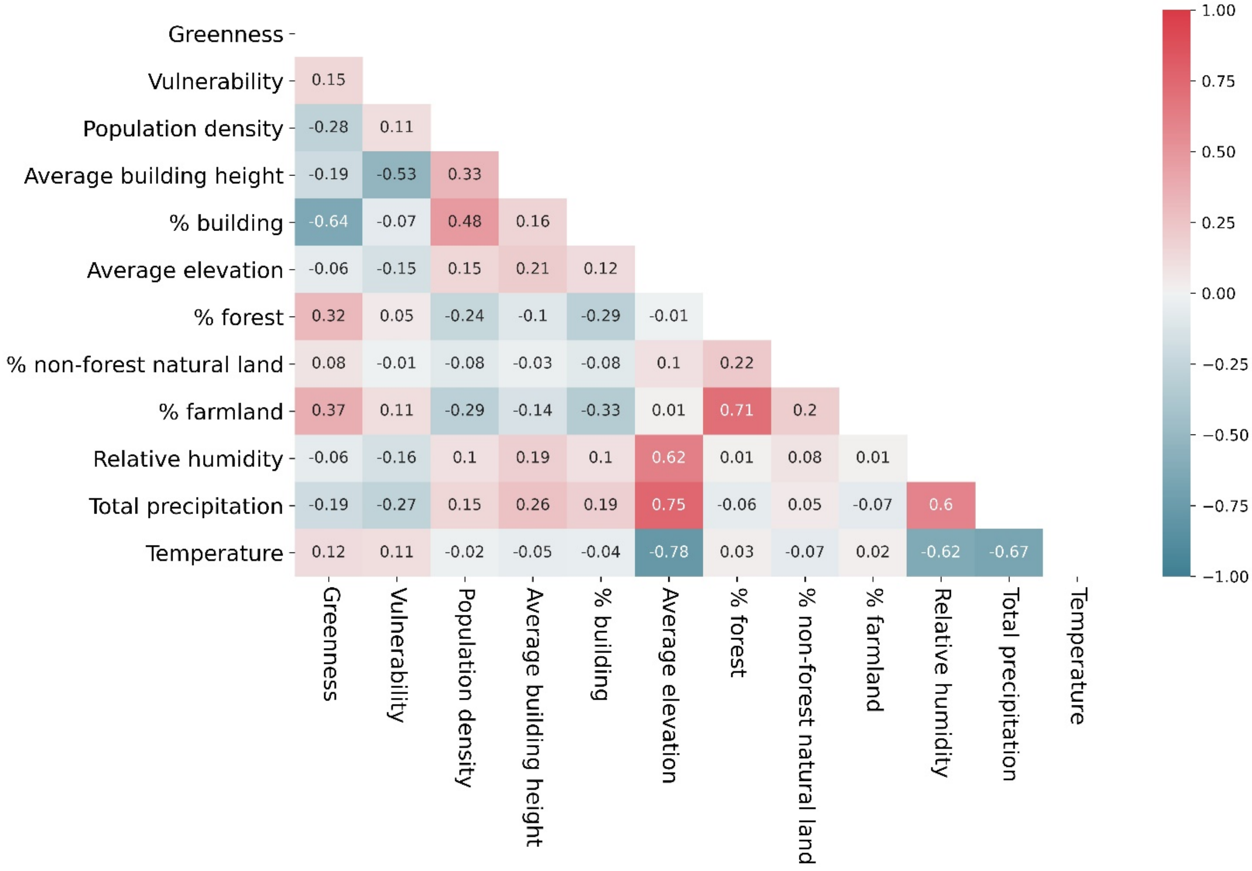

Figure S1 Spearman's rank correlation between the exposure and covariates.

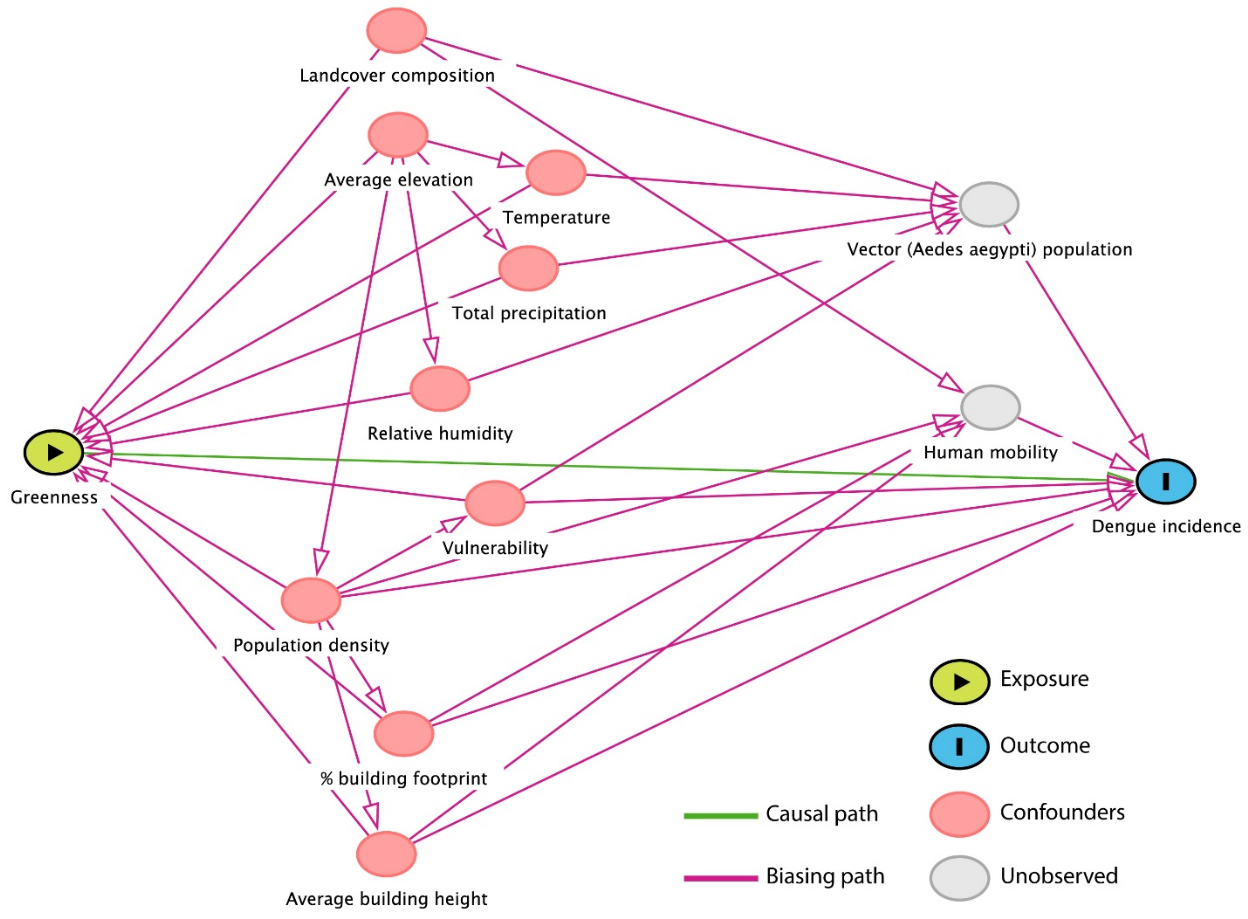

Figure S2 Directed acyclic graph for the outcome, exposure, and confounders

Table S1 Sensitivity test with alternative model specifications

Sensitivity test for model 2, Table 3

	Main model	Alternative specifications		
	(2)	(2-1)	(2-2)	(2-3)
Fix effects				
Greenness (-10-10)	0.949	0.812***	0.949	0.980
	[0.878,1.025]	[0.718,0.919]	[0.890,1.011]	[0.904,1.061]
Vulnerability (HVI, 0-10)	1.254***	1.354***	1.254***	1.247***
	[1.162,1.354]	[1.219,1.504]	[1.193,1.319]	[1.139,1.364]
Controls	Yes	Yes	Yes	Yes
Random intercept	Yes	No	Yes	No
Random slope	No	No	No	No
Fixed effects	No	No	No	Yes
Confidence interval	Neighborhood-cluster-robust	Neighborhood-cluster-robust	Independent	Neighborhood-cluster-robust
Number of observations	3826	3826	3826	3826

Sensitivity test for model 3, Table 3

	Main model	Alternative specifications
	(3)	(3-1)
Fix effects		
Greenness (-10-10)	0.915**	0.915**
	[0.843,0.994]	[0.852,0.984]
Vulnerability (HVI, 0-10)	1.269***	1.269***
	[1.176,1.368]	[1.205,1.335]
Random effects [a]		
var(Greenness)	0.050***	0.050***
	[0.022,0.115]	[0.025,0.100]
var(Intercept)	0.689***	0.689***
	[0.420,1.129]	[0.448,1.059]
Controls	Yes	Yes
Random intercept	Yes	Yes
Random slope	Yes	Yes
Confidence interval	Neighborhood-cluster-robust	Independent
Number of observations	3826	3826

Sensitivity test for model 4, Table 3

	Main model	Alternative specifications
	(4)	(4-1)
Fix effects		
Greenness (-10-10)	1.316***	1.316***
	[1.108,1.564]	[1.169,1.483]
Vulnerability (HVI, 0-10)	1.824***	1.824***
	[1.509,2.205]	[1.609,2.069]
Greenness × Vulnerability	0.879***	0.879***
	[0.825,0.938]	[0.845,0.915]
Random effects [a]		
var(Intercept)	0.532***	0.532***
	[0.440,0.644]	[0.448,0.633]
Controls	Yes	Yes
Random intercept	Yes	Yes
Random slope	No	No
Confidence interval	Neighborhood-cluster-robust	Independent
Number of observations	3826	3826

Note: A coefficient, or incidence rate ratio (IRR), is the factor by which the dengue incidence rate (dengue cases per 100,000 residents) changes for a one-unit increase in the corresponding covariate, when holding other covariates constant. Vulnerability is measured by the Health Vulnerability Index (HVI) that encompasses indicators of health-related infrastructures and resident socioeconomic status from the 2010 census. Models additionally controls for population density, average building height, the percentage of census tract area covered by building footprints (% building), land cover composition (% forest, % non-forester natural land, % farmland), relative humidity, total precipitation, and temperature. The coefficients of these covariates are omitted from the table for display purpose *, **, and *** indicate significant at p-value < 0.10, p-value < 0.05, and p-value <0.01. 95% confidence intervals are in square brackets. Independent confidence intervals assume the census tracts are independent of each other. Neighborhood-cluster-robust confidence intervals accounts for the spatial autocorrelation between census tracts within the same neighborhood and heteroskedasticity.

[a.] Random effects are the variances of log transformed coefficients (IRRs).
